# Eurocan Platform meeting: European recommendations for biomarker-based chemoprevention trials

**DOI:** 10.3332/ecancer.2014.488

**Published:** 2014-12-08

**Authors:** Linda Cairns

**Affiliations:** Science Editor, *e*cancermedicalscience, European Institute of Oncology, Via Ripamonti 435, Milan 20141, Italy

**Keywords:** cancer prevention, biomarkers, eurocanplatform, prevention trials

## Abstract

Chemoprevention or the now more preferred ‘cancer prevention’ is the long-term administration of a biological or chemical agent to reduce the risk of cancer. This approach has long been active in individuals at high risk of developing breast or colon cancer. The aim of this expert meeting was to review the current status of the field of cancer prevention and potential, emerging biomarkers specifically focusing on breast, colon, and lung cancer but also with sessions on ovary and prostate.

Jack Cuzick started the meeting discussing the challenges in developing and validating biomarkers for cancer prevention. Breast cancer is escalating exponentially, and by 2020, there will be around 2 million new cases per year. There is an urgent need to identify high-risk populations and to identify minimally toxic preventative therapy. He discussed the known risk factors associated with breast cancer: family history, reproductive/hormonal factors and benign pathology. He touched on the genes that can predict risk, including the well-established BRCA1/2 as well as other more recent discoveries (Figure 1). He mentioned the importance of mammographic density as an individual risk factor, and this will be discussed later. Single nucleotide polymorphisms, frequently called SNPs, are the most common type of genetic variation among people and can be useful for refining risk estimates in women with phenotypic risk factors for breast cancer. Cuzick presented the results from two randomized tamoxifen prevention trials in which an SNP panel was included to refine risk estimates in women at high risk of breast cancer. However, the SNP profiles did not appear to predict benefit from tamoxifen. He pointed out that there is still a need for biomarkers for late recurrence in breast cancer. Finally, he discussed the need for a better biomarker than PSA for prostate cancer and discussed the use of cell cycle progression genes CCP, i.e., genes which encode products that are required during cell cycle progression. The predicted risk of death from prostate cancer based on the combined cell cycle clinical (CCR) score and the CAPRA score can be calculated.

Powel Brown followed on with a presentation on biomarkers for Chemoprevention Trials: Filling the Gap Between the Bench and the Clinic. In order to test effective cancer preventive interventions we need to test promising prevention agents in preclinical models and in human prevention trials, measure drug-response biomarkers and also to identify surrogate endpoint biomarkers in animals that can then be used in Phase I and II cancer prevention trials in humans. A number of risk biomarkers have been identified which can also be used to monitor response to preventive drug treatment. These currently include mammographic breast density, blood hormone, and insulin growth factor levels and breast tissue precancerous changes called hyperplasia (a buildup of normal-looking cells in breast ducts) and atypical hyperplasia (a buildup of abnormal but not cancerous cells in breast ducts).

Retinoids are promising preventive agents and *in vitro* studies of bexarotene indicate that they are able to inhibit pre-malignant and breast cancer cell growth. The activity of bexarotene in breast cancer prevention was assessed in a biomarker-modulation trial, however, there was no significant difference in change of Ki67 index between bexarotene and placebo arms. Now other rexinoids are being tested to develop more effective and safer preventive drugs.

Karen Brown went on to discuss the possibility of PARP inhibition as prevention. Breast cancer landmark studies have demonstrated the activity of PARP inhibitors in BRCA mutation carriers and in triple negative breast cancer. In fact, a PARP inhibitor biomarker-modulation trial is being planned. She concluded that future efforts should focus on developing accurate risk markers, drug-response markers, and surrogate endpoint biomarkers.

Eva Szabo‘s talk was titled ‘Phase I–II chemoprevention trials: lessons from the NCI consortia experience’. The goal of early phase trials is to identify the ‘signal’ and thereafter to try understand the mechanism of action and to select high-risk participants.

The NCI consortia have focused on intraepithelial neoplasia (IEN) as it is recognized as an identifier of high-risk population. She discussed the various Phase I and II trials and the information they provide. In imaging studies, which can be useful for monitoring response, e.g. CT-detected lung nodules, she pointed out that the problem is that if there is no biopsy there is no biological information.

PI3K is a potential Molecular biomarker for lung dysplasia as the PI3K pathway is activated in smokers with dysplasia in airway (p < 0.001). Myo-inositol inhibited PI3K activation in normal bronchial airways in smokers, resulting in regression of dysplasia.

Andrea De Censi discussed ‘Why the results of phase III trials have not been translated into clinical practice?’ To address this issue, several points were to be considered. First a prevention treatment has to be proposed only to high-risk subjects, it is not aimed at the general population.

There are four main barriers to the development of chemo prevention drugs:

1. Uncertainty of reimbursement for new agents
  Depending on the specific national health system the treatment may not be reimbursed, especially if it is an off label treatment (European countries except UK) or for new agents.
2. Limitations in current patent law and intellectual property protection
3. Limitations in emerging prevention science, evolving designs of clinical trials, and the process of drug approval
4. Limited public participation in clinical trials.

Certainly there is the need for more awareness starting from the physician to reach the target population. It is quite difficult and takes time to explain the concept of the individual risk to people. And once the increased risk has been defined, how much is the ‘minimum effect’ required to consider a proposed treatment valid (2%, 10% more)?

One criticism that the major prevention trials often come up against is that there is no evidence of mortality reduction. This is a debate which is difficult to resolve since in primary prevention the main aim is incidence, mortality is ‘too far away’ and would require countless subjects and years to be proven.

One more topic is the lack of commercial interest and consequently it is very difficult to perform phase III clinical trials. One short-term goal could be the identification of an interim sign of efficacy, in order to encourage the responding subjects to continue treatment and to stop the one that may not benefit the individual.

Probably the younger generation will be more favourable to prevention treatment.

Giancarlo Pruneri, in his talk on **DIN/DCIS: more shade than light**, discussed the fact that intra epithelia neoplasias present a problem in terms of diagnosis reproducibility especially for the DIN1a or flat epithelial atypia. In non-highly specialized centres the surgical strategies (DIN1a vs DIN1b) and the importance of the margins is still under discussion. Finally, the predictive and or prognostic factors are still unclear compared to other more invasive tumours.

It is important to recognize and distinguish types of DIN that tend to recur from those that do not. Multigene expression assays can be helpful in scoring these lesions into categories that predict recurrence risk. However, they do not appear to be significantly better compared to the standard biological assays carried out using standard immunohistochemistry.

Proliferation matters: the ‘oncotype DX’ is an assay that harnesses the power of genomics to provide a more precise and accurate assessment of risk based on individual tumour biology. It encompasses a panel of 21 genes, many being proliferation genes within the tumour to determine a Recurrence Score. However, Ki67 expression may be just as informative and especially as DIN characterization has to be reproducible in all hospitals and not only in highly equipped labs, KI67, which is affordable worldwide, may be more appropriate.

It is important to improve the reproducibility of Ki67 and this is a work in progress. We have shown that it was possible to train laboratories around the world using ‘web-based standard images’. After the training, the reproducibility among labs showed a significant trend for improvement.

We have also shown that Ki-67 can be a good indicator of which patients with DIN2 may benefit from radiotherapy (RT). In patients with DIN2 with necrosis who underwent breast conservative surgery followed by RT versus no RT gave an HR of 0.18 (95% CI 0.07–0.46) whereas no difference was observed in the patients with a ki67 of <14%.

Andrea De Censi discussed cancer adjacent intraepithelial neoplasia (IEN) as a target biomarker for screening new preventive agents

The concept of the ‘cancerization field’ in mammary carcinogenesis postulates that around the tumour there are already present cells with genetic and epigenetic alterations that may cause a subsequent second cancer. The pre-surgical window of opportunity (WOP) trials is a powerful tool to test new and old drugs, not only on the tumour tissue, but also on adjacent tissue.

The post-treatment tumour expression of Ki-67 has been shown, by the group of decency and by other groups to be correlated with disease-free survival and overall survival. Evaluated Ki-67 in adjacent IEN or atypical hyperplasia (AH) can be used as a biomarker for preventive activity. Two trials have shown that Lapatinib and Metformin decreased Ki-67 in both IEN and AH. Metformin seems particularly effective in HER2+ve and ER+ve subgroup.

Patrizia Pasanisi discussed the use of ‘Hormones and Growth factors as markers of risk and prognosis in breast cancer’. Glucose levels, glycemic index, etc. have been associated with an increased breast cancer risk. This hypothesis has prompted research on dietary carbohydrates and their possible relationship to breast cancer risk. High glucose levels are associated with increased risk of breast cancer. Patrizia’s group assessed the effect of peri-diagnostic fasting blood glucose in women treated for BC in order to see if there is a link between serum glucose and prognosis. They retrospectively investigated 1,261 women diagnosed and treated for stage I-III BC at the National Cancer Institute, Milan, in 1996, 1999, and 2000. The risk of recurrence death was significantly higher in the increased glucose group compared to the reference. The increased risks remained for a follow-up of 9.5 years [1].

Since a possible way for carbohydrates to be linked to breast cancer risk is through their effect on insulin levels, several lines of evidence suggest that insulin levels may directly or indirectly affect breast cancer risk.

The association between obesity and breast cancer is in part related to oestrogen which is synthesized by adipose tissue in post-menopausal women. Oestrogen has long been a known risk factor for breast cancer. Obesity is also associated with high levels of insulin. Gunter at al demonstrated that hyperinsulinemia is an independent risk factor for breast cancer in post-menopausal women.

Carbohydrates in our diets affect the body’s levels of blood glucose (blood sugar) and the hormone insulin. In post-menopausal women there is a correlation between high levels of oestrogen and androgens and breast cancer, whereas in pre-menopausal women only testosterone is associated. Three prevention trials were discussed the Cos2 (increasing activity), the DIANa-5 and the MeMeMe.

Jack Cuzick gave a second presentation on the **role of mammographic density in risk prediction and response to treatment**. Increased mammographic density is associated with a markedly increased risk of invasive breast cancer. In a study nested within the IBIS, a randomized prevention trial of tamoxifen versus placebo, Cuzick and colleagues [2] showed that, compared with all women in the placebo group, those in the tamoxifen arm who experienced at least a 10% reduction in breast density had a 63% reduction in breast cancer risk. However, those who took tamoxifen, but had a reduction in breast density of less than 10% had no risk reduction. In the placebo arm, breast cancer risk was similar in subjects who experienced less than a 10% reduction in PMD and those who experienced a greater reduction. So the change in breast density in the 12–18 months after starting treatment is an excellent predictor of response to tamoxifen in the preventive setting.

Dorella Franchi spoke about **biomarkers of risk for ovarian cancer**. Of all inherited ovarian cancers, BRCA2 is involved in 45% of cases. More than two out of three cases of ovarian cancer (Ov Ca) are diagnosed at an advanced stage (stage III-IV) and as survival is inversely correlated with clinical stage there is an urgent need for early diagnosis. The only real biomarker in Ov Ca screening is CA125. It is elevated in 47% of women with early-stage Ov Ca and in 80-90% of advanced stage Ov Ca. However, numerous, non-neoplastic conditions can cause an elevation in CA125 levels and so by itself is not a reliable biomarker. Furthermore, when used in combination with transvaginal ultrasound compared with usual care did not reduce ovarian cancer mortality. The only real preventative measure for individuals at high risk of developing OvCa is risk-reducing salpingo-oophorectomy (RRSO). It has been shown that in 122 BRCA1/2 women undergoing RRSO (5.7%) were found to have an early malignancy which originated in the distal fallopian tube.

The human epididymis protein 4 (HE4) measurements in serum have been proposed for improving the specificity of laboratory identification of OvCa. The combination of HE4 and CA125 (ROMA: Risk of Ovarian Malignancy Algorithm) showed the highest sensitivity relative to any other dual combination of markers. It represents a potential tool to triage women with a pelvic mass to the appropriate treatment. Other serum biomarkers under evaluation were discussed, but these still need to be validated. To date, the best known chemoprevention agent for OvCa is the oral contraceptive, which gives a 46% risk reduction of ovarian cancer in the general population.

Dominique Scherer then talked about **genetic markers for CRC prevention**. Risk alleles exist, but they are not sufficient to predict individual risk. Chemoprevention strategies include aspirin; 25% reduction in the incidence and 31% reduction in mortality. However, this benefit is only in patients with a PI3K. They are using a genotyping approach to subdivide the population into those who will benefit, but without the toxic side effects. Along with De Censi they are involved in the ASMET trial combining Aspirin and Metformin in the prevention of CRC. The aim is to identify tissue and circulating biomarkers.

In conclusion, the heritable component of the CRC is not completely elucidated. Aspirin/NSAIDs have great potential for chemoprevention of CRC. The interaction between genetics and prevention strategies need to be considered and further investigated for personalized prevention strategies.

Adriana Albini discussed the search for biomarkers of cancer angioprevention for clinical studies. Angiogenesis is an early event in multistage tumorigenesis and so needs to be prevented. She discussed some potential molecules for antiangiogenic treatment.

Her group has recently discovered a tumour infiltrating NKs as a potential marker and a target for therapeutic and preventive approaches [3]. The idea is to use these NK cells to attack the tumour.

Ugo Pastorino discussed **State-of-the-art and future perspectives in lung cancer chemoprevention**. The first-generation lung cancer chemoprevention trials, including the ATBC lung cancer prevention study were limited due to the over simplistic interpretation of epidemiological data. These trials also suffered from the lack of intermediate biological endpoints and the exposure to known carcinogens within the trials. Second-generation chemoprevention trials need to be designed with more active and less toxic agents and the use of reliable biomarkers. The participants should be high-risk individuals. He discussed extensively the use of microRNAs as predictive and diagnostic markers of lung cancer and their use in improving the efficacy of screening. The only potentially active agent for chemoprevention in smokers is verenicline, whereas aspirin, metformin and miRNA modulators may be conceivable in former smokers. In the end, the best prevention for lung cancer incidence is to stop smoking.

Fabrizio Bianchi gave us an introduction to microRNAs and the clinical validity of a microRNA-based blood test in lung cancer. miRNAs can act as either ‘downregulators’ or ‘enhancers’ of gene expression. He explained how miRNAs are deregulated in cancer and gave some examples of how miRNAs might induce tumour-promoting effects. miRNA secreted by cancer cells might affect the translational profile of surrounding normal cells or render self-promoting effects.

The increase of tissue-specific miRNA in the blood and plasma of individuaks with tumours supports the use of miRNA as ‘disease biomarkers’. The main features arguing for the suitability of miRNAs as ‘liquid biomarkers’, i.e. stability and tissue-specific expression were discussed. Early detection of lung cancer is the main strategy to improve disease prognosis. Early diagnosis can be achieved with tomography-based screening in high-risk individuals, i.e. the COSMOS in IEO. However, concerns remain regarding the feasibility of large-scale population screening. The group of Bianchi has developed a blood test, based on the detection of serum miRNAs, that identifies early-stage non-small cell lung carcinomas (NSCLCs) in a population of asymptomatic high-risk individuals with ~80% accuracy. The test is based on the detection of 34 miRNAs. The test displays a number of features of clinical relevance that project its utility in programmes for the early detection of NSCLC and is being validated within the COSMOS II trial.

Hector Hernandez-Vargas discussed Epigenetics as a source of prevention biomarkers. In particular, he is looking at DNA methylation as a biomarker in cancer prevention. An ideal biomarker should be able to predict an outcome with high sensitivity and specificity. In addition, it should be cost-efficient and not invasive. DNA methylation is a good as it is stable, its reported deregulation at early stages and its accessibility. However, there is still no widespread DNA methylation biomarker for cancer prevention and we still need to wait for new data coming from genome-wide analysis (GWAS).

Phillipe Autier spoke about **biomarker for the identification of men at high risk of aggressive prostate cancer: the past**. Similarly, to other cancers in the screening age, we are observing a steep increase of incidence and a total stable percentage of mortality. Out of seven men with prostate cancer only one will die from it.

PSA is currently the best biochemical marker for prostate cancer. More than the absolute concentration, its biological variation (within subject) can help to beter determine cancer risk, a single measurement can lead to more unnecessary biopsies. But PSA does not help to discriminate cancer aggressiveness.

The PLCO Cancer Screening Trial concluded that there was no evidence of a mortality benefit in the screening intervention group.

Massimo Maffezzini talked about **new avenues for biomarkers of potentially deadly prostate cancer. Which kind of tumour do we want to prevent?**

The screening for prostate cancer has increased to nearly 50% of the diagnosis to low-grade cancer, does this represent success or over-diagnosis? Is the current treatment of low-grade prostate cancer, in fact, over treatment? Clearly there are prostate cancers that won’t cause disease-specific morbidity nor mortality during the patient’s life. Several clinical trials are ongoing to address this issue. The (short-term follow up of the) PRIAS study indicates that active surveillance is a reasonable strategy to reduce overtreatment.

A greater research effort has to be focused on risk subjects stratification for a more personalized surveillance and intermediate biomarkers to test new preventive agents. Recognized and easily assessed risk factors are: age, race, family history, obesity, boldness, and PSA; new biomarkers under evaluation: Single nucleotide polymorphisms (rs 11672691), Cell Cycle Progression Score, miRNA (-23b/-27b).

Active prevention is possible and so far the more effective agents are the 5-α reductase inhibitors. Non-steroidal anti-inflammatory drugs (NSAIDs and in particularly aspirin), SERMs, iNOS inhibitors, cell cycle blockers and apoptosis inducer are also potential agents to be studied in prostate cancer prevention.

## Conclusion

The aim of this meeting was to establish guidelines for biomarker discovery and validation relative to high-risk individuals. Five types of biomarkers were outlined:

(a) Prognostic
(b) Predictive
(c) Risk biomarkers
(d) Drug-response biomarkers
(e) Diagnostic biomarkers

Screening and biomarker discovery will be important in distinguishing aggressive from less aggressive disease. This is something that needs more work.

To test effective cancer preventive interventions, these agents need to be tested in several preclinical models and in human prevention trials. The use of clinically achievable concentrations of agents is essential in preclinical models. It will be necessary to chronically expose *in vitro* systems and cells that represent precursor states to the agents under study. It will be important to measure drug-response biomarkers to assess effects of the drug in blood and tissue. Surrogate endpoint biomarkers need to be tested in animals that can then be used in Phase I and II cancer prevention trials in humans.

In human prevention trials, Phase 3 trials need to demonstrate a reduction in mortality. They should also validate the surrogate biomarker. Biomarkers should be developed at the time of the clinical trial. It will also be important to show that the marker is efficacious in the (original) animal model. A biomarker should demonstrate an effect of the intervention on carcinogenesis-relevant pathways. Biopsies are necessary to understand the biology.

Future studies will require multiple biomarkers, a single biomarker would be too naïve in a multifaceted disease. Circulating Free DNA has great potential as a surrogate tumour marker for monitoring efficacy and personalizing interventions.

Finally, there was consensus that we need more money from the EU for clinical prevention trials. The proposal is to put forward a Horizon 2020 project to discover effective preventative agents and eventually an ERANET. There is a need to nest prevention into screening programs.

## Figures and Tables

**Figure 1.**
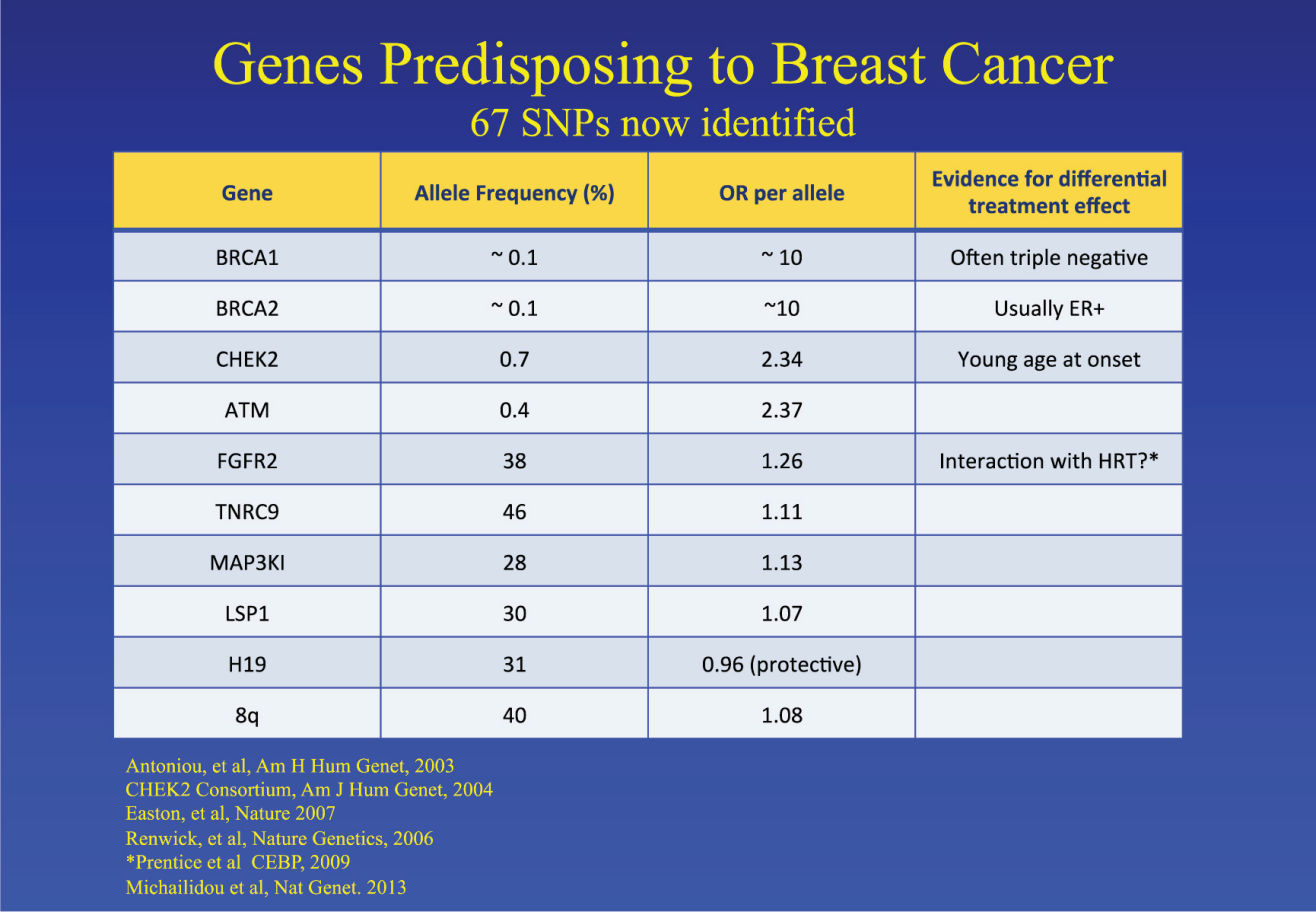
Genes which predispose to an increased risk of breast cancer [4–9].
